# Artificial Intelligence Approaches to Predict Postoperative Length of Hospital Stay in Head and Neck Cancer Patients: A Systematic Review †

**DOI:** 10.3390/diagnostics16020263

**Published:** 2026-01-14

**Authors:** Willian Nogueira Silva, Anna Luíza Damaceno Araújo, Alvaro Sanabria, Ludhmila A. Hajjar, Juan Pablo Rodrigo, Karthik N. Rao, Ewa Florek, Remco de Bree, Alfio Ferlito, Luiz Paulo Kowalski

**Affiliations:** 1 Head and Neck Surgery Department and LIM 28, University of São Paulo Medical School, São Paulo 05403-900, Brazil; willian.nogueira@fm.usp.br (W.N.S.); luiz.kowalski@hc.fm.usp.br (L.P.K.); 2 Hospital Israelita Albert Einstein, São Paulo 05652-900, Brazil; 3 Department of Surgery, School of Medicine, Universidad de Antioquia, Medellin 050015, Colombia; alvarosanabria@gmail.com; 4 Department of Cardiopneumology, InCor, University of Sao Paulo Medical School, São Paulo 05403-900, Brazil; ludhmila@usp.br; 5 Department of Otolaryngology, Hospital Universitario Central de Asturias, University of Oviedo, ISPA, IUOPA, CIBERONC, 33011 Oviedo, Spain; jprodrigo@uniovi.es; 6 Department of Head and Neck Oncology, All India Institute of Medical Sciences, Raipur 492099, India; karthik.nag.rao@gmail.com; 7 Laboratory of Environmental Research, Department of Toxicology, Poznan University of Medical Sciences, 60-631 Poznan, Poland; eflorek@ump.edu.pl; 8 Department of Head and Neck Surgical Oncology, University Medical Center Utrecht, 3584 CX Utrecht, The Netherlands; r.debree@umcutrecht.nl; 9 International Head and Neck Scientific Group, 35128 Padua, Italy; profalfioferlito@gmail.com; 10 Department of Head and Neck Surgery and Otorhinolaryngology, A.C. Camargo Cancer Center, São Paulo 01509-010, Brazil

**Keywords:** artificial intelligence, machine learning, predictive models, length of stay, head and neck surgery

## Abstract

**Background/Objectives:** The aim of the present systematic review is to evaluate the performance of AI models for length of stay prediction. **Methods:** This SR was carried out in accordance with PRISMA 2020 and registered in PROSPERO database (CRD420251039985). Using the PICOS framework, we formulated the following research question: “Can artificial intelligence models accurately predict hospital length of stay (LOS) in patients undergoing head and neck (H&N) cancer surgery?” We searched the Cochrane Library, Embase, PubMed, and Scopus, with additional gray literature identified through Google Scholar and ProQuest. Risk of bias (RoB) was assessed using the Prediction Model Risk of Bias Assessment Tool (PROBAST), and a narrative synthesis was performed to summarize qualitative findings. **Results:** Of 1304 identified articles, 5 met inclusion criteria, covering 5009 patients. All studies used supervised learning to predict LOS with different variables presenting stronger associations with increased hospital LOS. Age, race, ASA score, BMI, and comorbid factors like smoking and arterial hypertension were comon variables across studies but not always the ones most strongly associated with LOS. One study also predicted discharge to non-home facilities and prolonged LOS; only one applied data balancing. Model accuracies ranged from 0.63 to 0.84, and area under the receiver operator characteristics curve (AUROC) values from 0.66 to 0.80, suggesting moderate discriminative performance. All studies had a high risk of bias, though no applicability concerns were noted. **Conclusions:** AI models show potential for LOS prediction after H&N cancer surgery; however, an elevated RoB and methodological shortcomings constrain the current evidence. Methodological improvements, external validation, and transparent reporting is essential to enhance reliability and generalizability, enabling integration into clinical decision-making.

## 1. Introduction

Historically, hospital stays were often longer in the past than they are today. In the past decades, hospitals were the primary setting for the treatment of chronic and acute illnesses. During this period, the length of stay (LOS) was important in determining the efficient use of hospital resources and planned bed occupancy. In recent years, LOS has become even more important due to changes in the healthcare system and growing concerns about the cost, capacity, and quality of care, with an increasing focus on reducing unnecessary hospital stay, readmissions, and complications related to prolonged LOS. Although LOS has always been an important variable in resource management and care planning, its meaning and impact have evolved over time, reflecting changes in healthcare practices and related policies. This means that increases in LOS directly affect the patient turnover cycle, leading to bed congestion and difficulties in resource management [1].

LOS therefore represents a key factor across multiples areas of medicine, including both clinical and surgical settings. The prediction of LOS has exciting potential to significantly improve care planning, pragmatically organize the allocation of different resources, schedule procedures, reduce surgical waiting times, and improve the organization of bed occupancy [2]. LOS directly influences bed occupancy and, consequently, the dynamics of hospital capacity [3]. In other words, inefficient management of patients occupying beds may result in increased waiting times for surgical procedures. Therefore, strategies for predicting LOS can be used to better understand this variable and reduce its random component, making it a crucial factor in care planning, as noted by Goshtasbi et al. [4].

Variable prediction plays a leading role across multiple fields, particularly in medicine, and represents an important strategy for improving variable understanding, guiding clinical decision-making, and increasing the information available to both clinicians and patients. Researchers apply multiple techniques for this purpose, ranging from quantitative methods and systematic data analysis to, more recently, artificial intelligence (AI) techniques. This approach offers great advantages, since the efficiency and high speed of processing enormous amounts of data, predictive accuracy, automation, continuous learning, and adaptability ensure greater sophistication in understanding these variables. AI’s ability to create specific models after processing data means that its predictive performance is related to the quality and volume of the training data. In other words, an algorithm refines and improves when presented with new datasets, thus driving this new component of modern medicine. As described by Kaul et al. [5], AI is currently capable of evaluating more complex problems through self-learning and enhances clinical practice to improve diagnostic accuracy and workflow efficiency.

This systematic review (SR) aimed to evaluate and synthesize evidence on the performance of AI models for predicting postoperative hospital LOS in patients undergoing H&N cancer surgery. Specifically, it sought to describe the types of AI methods applied, the input variables and modeling strategies used, the predictive performance reported, and the limitations identified in the development and validation of these models.

In this study, we use the term Artificial Intelligence (AI) as an umbrella concept that encompasses computational techniques outside the simulation of intelligent behaviors. Machine Learning (ML) functions as a subset of AI, focusing on algorithms that learn from data. When we refer to Deep Learning (DL), we are specifically referring to architecture based on deep neural networks, used in scenarios with large volumes and complex data.

## 2. Methods

### 2.1. Eligibility Criteria

The focused review question—“Can artificial intelligence models accurately predict hospital LOS in patients undergoing H&N cancer surgery?”—and the eligibility criteria were derived based on the PICOS criteria. Participants/population consisted of individuals diagnosed with H&N cancer who underwent surgery in any modality. The intervention involved artificial intelligence (i.e., ML/DL prediction models). The primary outcome was postoperative LOS, and only studies that developed or validated prediction models for LOS were included. Studies were retrospective cohort studies that developed and validated AI-based prediction models. The included articles should provide a detailed description of the input variables (i.e., patient and clinical characteristics available preoperatively and/or intraoperatively), preferably highlighting the features most strongly associated with the outcome. Any type of AI prediction model was considered, including linear or non-linear ML algorithms, DL models, and ensemble methods. Multivariable linear regression models could also be included if they are used for predictive purposes and integrated into automated pipelines, defined as models integrated into algorithmic workflows for outcome prediction rather than solely for inferential statistical analysis. Excluded studies were those that did not predict LOS, included other types of cancers or diseases, employed classical statistical models (e.g., multivariable linear regression aimed at understanding relationships between variables, emphasizing interpretation, statistical significance, and assumptions), focused on predicting other outcomes (e.g., acute kidney injury, thrombosis, embolism, pneumonia, among others), or investigated treatment modalities other than surgery.

### 2.2. Information Sources and Search Strategy

Tailored search strategies were conducted on 5 November 2024, across the following electronic databases: Cochrane Library, Embase, PubMed, and Scopus. Gray literature was explored through Google Scholar and ProQuest. Additionally, the reference lists of included studies and relevant SRs were manually screened to identify any potentially eligible records not captured by the database searches. The complete search strategy is shown in Supplementary Materials.

### 2.3. Selection Process

Duplicate records were initially removed automatically using Rayyan [6]. Following deduplication, two reviewers (W.N.S. and A.L.D.A.) independently screened titles and abstracts in the first phase of study selection. Eligibility criteria were then applied to the articles retained for full-text review. Any disagreements were assessed by a third reviewer (L.P.K.) and resolved through consensus. Rayyan was used both for duplicate removal and for independent screening of titles and abstracts by two reviewers.

### 2.4. Data Collection Process and Data Items

Data extraction was performed by one reviewer (W.N.S.) and subsequently cross-checked by a second reviewer (A.L.D.A.). The variables to be extracted from the included studies were predefined by the authors as follows: author, year, type of cancer/anatomical site, number of patients, patient distribution for training/validation, date range, learning modality, predicted outcomes, type of data, input variables, mean LOS, feature selection method, AI models, performance metrics as accuracy and area under the receiver operator characteristics curve (AUROC), variables most associated with LOS, study limitations, and study conclusion.

### 2.5. Risk of Bias (RoB) Assessment

Each study was independently assessed by two authors (W.N.S. and A.L.S.O.) using the Prediction model Risk of Bias Assessment Tool (PROBAST) to evaluate the risk of bias (RoB) and applicability of diagnostic and prognostic prediction model studies [7,8].

### 2.6. Effect Measures

Accuracy and AUROC were selected as primary metrics due to their common use in the LOS prediction literature. While AUROC is less susceptible to class imbalance than accuracy and assesses model discrimination, it can still be misleading with skewed datasets [9].

The data were synthesized using a combination of both qualitative and quantitative methods, providing a comprehensive overview of the evidence. Due to substantial heterogeneity in study design, modeling approaches, input variables, and performance metrics, a quantitative meta-analysis was not attempted, and a narrative synthesis was considered the most appropriate approach.

## 3. Results

### 3.1. Study Selection

**Figure 1:**
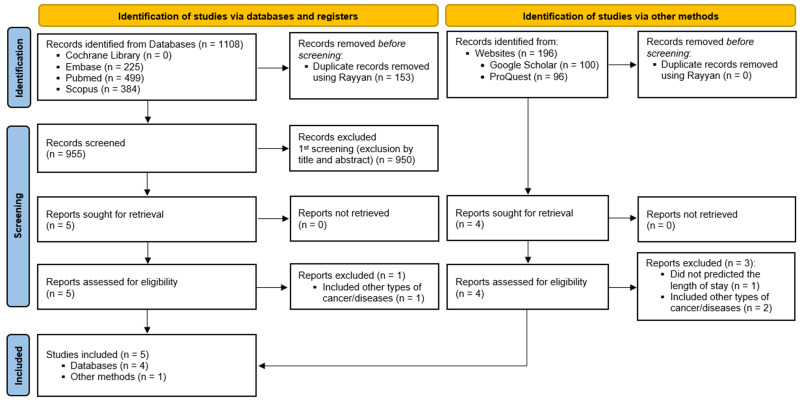
PRISMA Flowchart from: [14]. For more information, visit http://www.prisma-statement.org/ (accessed on 30 June 2025).

Among a total of 1304 records identified through the search strategy, 5 articles [4,10,11,12,13] fulfilled the eligibility criteria and were included in this SR. The study selection process is outlined in the PRISMA Flowchart (Figure 1), and the reasons for excluding each article reviewed in full text during the second phase are detailed in Supplementary Materials.

### 3.2. Study Characteristics

A total of 5009 patients from 5 studies [4,10,11,12,13] were included. Admission dates ranged from 2005 to 2022 covering an interval of at least two years [12] and at most twelve years [4]. Surgical procedures varied from complex H&N surgery [4,13], free flap reconstruction [11,12] and vestibular schwannoma resection [10].

All studies implemented supervised learning models to predict LOS as the main outcome, with one study also predicting discharge to nonhome facility (DNHF) [4], and any LOS above the median length of stay [prolongued lenght of stay (PLOS)] [11]. For this matter, four studies utilized pre, intra and postoperative data [10,11,12,13] while one utilized only preoperative data [4]. The input variables, feature selection methods, AI models with respective performance metrics, and variables most associated with LOS are listed in Table 1. Only one study applied the Synthetic Minority Oversampling Technique (SMOTE) to address data imbalance [13].

Several variables included in the models were similar across studies, such as age, race, ASA score, BMI, and comorbid factors like smoking and arterial hypertension. However, each study identified different variables with stronger associations with increased hospital LOS. Preoperative transfusion, elective surgery, procedure type, coronary artery disease, hypertension, ASA score, ischemia time of the graft, transplant (microvascular/local flap), baseline creatinine, surgery duration, sex, age, BMI, albumin, hemoglobin, and smoking status were the most prevalent predictors identified, respectively, across different studies. Overall, there was a consistent effort to investigate associations with important medical characteristics such as age, race, BMI, laboratory data, and comorbidities. However, these variables were not always the ones most strongly associated with LOS.

Limitations of the included studies were pointed pointed out as retrospective design and single-center data (limiting generalizability and introducing bias) [4,10,11,12,13], no external validation/only internal validation (raising risk of overfitting) [4,12], limited or modest sample size for ML modeling [4,10,12], lack of specific or comprehensive clinical variables (e.g., absence of otorhinolaryngological variables in Goshtasbi et al. [4] and unmeasured socioeconomic/logistic factors in Namavarian et al. [12]), potential selection bias (unclear inclusion/exclusion, data exclusions) [4,10], heterogeneous or poor model performance [13], limited generalizability due to focus on specific procedures [13], limited interpretability of models [12], inadequate handling of data or modeling limitations [4,12], potential missing important intraoperative data/time series being underexplored [11], reduced statistical power due to data splits or missing data [11,12], need for prospective, multicenter studies with larger samples [11,12,13].

### 3.3. RoB in Studies

In the patient domain, all studies presented low RoB since all inclusions and exclusions were appropriate and the data sources were from cohorts.

Four studies [10,11,12,13] presented a high RoB in the predictor’s domain, which was recorded because not all predictors were available at the time the model was intended to be used. This means that some predictors used to build the models were not actually available at the point when the prediction needed to be made in practice (i.e., preoperatively) leading the models to rely on future or unavailable information. Importantly, the reliance on postoperative predictors fundamentally limits the clinical utility of these models for preoperative planning, which represents a primary motivation for LOS prediction in surgical decision-making. Thus, following the TRIPOD criteria, although understanding these correlations after the surgical procedure is of great importance, using postoperative data at the time of model development intended to predict LOS introduces bias.

For the outcome domain, all studies presented low RoB since the outcome was determined appropriately, a pre-specified or standard outcome definition was used, predictors were excluded from the outcome definition, the outcome was defined and determined in a similar way for all participants, the outcome was assessed without knowledge of predictor information, and the time interval between predictor assessment and outcome determination was appropriate.

All studies presented high RoB in the analysis domain, which was registered if univariable analysis is used to select predictors [4], if the relevant model performance measures were not evaluated appropriately [10,11,12,13], which creates a significant difficulty in comparing the articles to reach a clearer consensus and understanding of the approaches and the actual performance of the models, or even in including them in meta-analyses; and if model overfitting and optimism in model performance were not accounted for, two common problems in AI-based studies protocols [9,15].

Therefore, a high RoB was identified in all studies (Table 2). The analysis raised no applicability concerns.

## 4. Discussion

### 4.1. Main Findings

Predicting LOS using AI in patients undergoing H&N cancer surgery has immense potential to improve care planning and resource allocation. This SR highlights the relevance of the topic and underscores important methodological limitations in the studies analyzed. Therefore, this SR aims to critically assess and synthesize existing studies on LOS prediction, particularly in H&N surgical patients, in order to identify the most effective approaches, to assess and summarize the predictive performance reported, to identify methodological limitations in the development and validation of these models, and highlight potential gaps that can inform future development of intelligent bed management systems and improve healthcare planning.

### 4.2. Comparison with the Existing Literature

In recent years, several SRs have addressed the application of predictive models in H&N surgery, but with different scopes. For example, Adeoye et al. [16] analyzed the impact and utility of ML-based prediction tools for cancer outcomes, focusing on low- and lower-middle-income countries and considering cancers in general, with unsatisfactory results for the models so far. Aly et al. [17] identified outcome prediction model studies, assessed their methodological quality, and evaluated their potential utility for clinical practice, focusing specifically on H&N squamous cell carcinoma and highlighting the elevated risk of bias. Buttigieg et al. [1] aimed to identify and summarize empirical research on the various variables that directly or indirectly impact LOS within tertiary hospitals to develop a LOS causal systems model. Moharrami et al. [18] evaluated the performance of ML models in predicting post-treatment survival and disease progression outcomes, including recurrence and metastasis, in H&N cancer using clinicopathological structured data. Like findings in other surgical fields, AI-based LOS prediction models in head and neck surgery show moderate performance but face common limitations such as heterogeneity, limited external validation, and restricted clinical integration. This indicates that the challenges observed are shared across surgical specialties rather than procedure-specific.

### 4.3. Methodological Considerations

Multiple variables across different models, such as age, race, ASA, BMI, and comorbid factors including smoking and arterial hypertension, showed a high correlation with LOS. However, each study identified variations in stronger associations with increased LOS. Preoperative transfusion, elective surgery, procedure type (resection), disseminated cancer, and history of congestive heart failure emerged as key factors in the analysis by Goshtasbi et al. [4]. Coronary artery disease and hypertension were most important in Dang et al. [10]. Operation time, ischemia time, transplant, ASA score, intensive care stay, and TNM stage were key predictors in a study of Vollmer et al. [13]. Age, baseline creatinine, monocyte count, duration of surgery, patient comorbidities, advanced disease state, prior treatments, resident teaching, surgeon skill, hospital volume, and anesthesia-related morbidity were main correlates of LOS increase in a study of Namavarian et al. [12]. Smoking status, hypertension, albumin, hemoglobin, intraoperative red blood cell transfusion, and intraoperative fresh frozen plasma transfusion were the most important features identified in study of Liu et al. [11]. The results from other studies include the following: fully dependent functional state (OR: 32.62), Black or African American race (OR: 1.75), and operating time (OR: 1.15) in Helman et al. [19]; age, diabetes, ASA physical status, Charlson comorbidity index, and repeat procedures in study of O’Brien et al. [20]; and sex, race, BMI, acute or chronic condition, emergency status, and ASA classification in study of Mason et al. [21]. The reviewed studies highlight a wide range of clinical, demographic, and surgical factors associated with increased LOS. Variables such as age, comorbidities (hypertension, coronary artery disease, congestive heart failure), functional status, surgical complexity, and laboratory markers consistently show significant correlations with LOS.

However, each study emphasizes different key predictors based on their specific population and context, indicating that no single universal set of factors can fully explain LOS variability, and training datasets often overrepresent individuals from specific geographic, ethnic, or socioeconomic groups, limiting the generalizability of the algorithm [22]. For instance, Goshtasbi et al. [4] underscore the importance of preoperative transfusion and procedure type, while Namavarian et al. [12] include surgeon skill and hospital volume as relevant contributors. This variability suggests that predictive models for LOS require tailoring to the clinical and institutional context, incorporating both objective clinical variables and structural or procedural factors. Additionally, sociodemographic factors like race and functional dependency appear to influence LOS, highlighting the role of social determinants in patient outcomes and reinforcing the need for multidimensional approaches in LOS prediction and management. Also, the type of healthcare system and assurance may affect LOS. Differences in surgical type and healthcare systems likely contribute to LOS variability, reinforcing the need for institution-specific model development and validation. There seems to be a need for more robust (recently found) predictors to include in these models, and, in addition, perioperative-pain- and opioid-related adverse effects, which have been associated with increased hospital LOS and costs in previous studies, were not systematically included in the reviewed models and may contribute to unexplained LOS variability. Multiple studies showed an association between low skeletal muscle mass and/or hand grip strength (sarcopenia) with increased LOS in patients undergoing major H&N surgery [23]. Pre-operative neutrophil-to-lymphocyte ratio and frailty also predict LOS [24,25].

The studies employed a wide range of statistical methods for feature selection, reflecting both the complexity of modeling LOS and the lack of consensus on a single best approach. Others, such as Goshtasbi et al. [4], began with univariate analyses to screen potentially relevant variables before using multivariate regression with stepwise elimination guided by the Akaike information criterion, balancing statistical significance and model parsimony. Others, such as Dang et al. [10] and Namavarian et al. [12] incorporated more advanced ML techniques, including random forests and LASSO regression, to rank variable importance and improve predictive performance. The consistent finding that ensemble models (random forests, gradient boosting machines) outperformed traditional linear regression underscores the limitations of linear approaches in capturing complex, non-linear relationships among predictors. Additionally, the use of tools like CHAID and SMOTE highlights efforts to manage variable interactions and class imbalance, further illustrating how diverse methodological choices can affect the identification of key predictors and model accuracy.

In addition to the heterogeneity of variables and methods, another crucial point is the lack of robust external validations and the low standardization in the evaluation of the performance of predictive models. Most studies focused on to internal validation (such as cross-validation), without assessing the applicability in independent cohorts, which compromises the generalization of results to different populations and clinical scenarios. In a subset of studies where external validations, multiple concerns emerge, and it can be another challenge to adopt AI algorithms for clinical evaluation [22]. Furthermore, although metrics such as RMSE, accuracy and AUC were reported, few analyses considered the clinical relevance of the predictions made, such as the impact of errors in bed allocation or perioperative management. This gap highlights the need for studies that integrate technical validation with real-world scenarios, addressing aspects such as model interpretability, integration with hospital systems, and practical utility for multidisciplinary teams. Furthermore, in class-imbalanced tasks such as LOS prediction, additional considerations such as precision, recall, F1 score, and Brier score supports a more robust performance assessment. Also, impairment curves can help verify confidence in probabilistic probabilities, an often overlooked but crucial aspect in clinical applications [26].

### 4.4. Strengths and Limitations of the Review

This systematic review has several strengths, including adherence to PRISMA guidelines, registration in PROSPERO, and the use of a standardized tool (PROBAST) to assess the risk of bias and applicability of prediction models. Additionally, this review focuses specifically on AI-based approaches for LOS prediction in head and neck cancer surgery, providing a focused synthesis of methods, variables, and model performance. However, important limitations should be acknowledged. The included studies were heterogeneous in surgical procedures, input variables, modeling strategies, and performance metrics, and most relied on retrospective, single-center data with limited or no external validation. These factors restrict the generalizability and clinical applicability of the reported models.

Beyond the limitations already discussed, several methodological challenges inherent to non-linear machine learning models warrant further consideration, including class imbalance, multicollinearity, non-linearity, overfitting, limited generalizability, and restricted interpretability in clinical prediction tasks [27]. Class imbalance, multicollinearity, and non-linear relationships among predictors are common in clinical datasets and may substantially affect model stability and performance. In addition, limited data quality and quantity, combined with high-dimensional feature spaces, increase the risk of overfitting, thereby restricting generalizability across institutions and patient populations. Another important limitation relates to model interpretability. Although non-linear models may achieve higher predictive performance, their limited transparency can hinder clinical adoption, particularly when decision-making requires clear justification. Approaches that enhance explainability, such as feature attribution methods, are therefore essential to support clinical trust. Furthermore, as highlighted by the lack of standardized benchmark datasets, the heterogeneity in data sources, variable definitions, and outcome reporting limits meaningful comparison across studies. This absence of benchmarking standards reinforces the need for harmonized data collection frameworks and external validation using diverse, multi-institutional datasets.

Future studies should prioritize prospective and multicenter designs, standardized reporting of performance metrics, inclusion of clinically relevant perioperative variables, and rigorous external validation across different healthcare settings to enhance robustness and facilitate translation into clinical practice. Due to substantial heterogeneity in study design, modeling approaches, input variables, and especially no performance metrics reported, a quantitative meta-analysis was not attempted, and a narrative synthesis was considered the most appropriate approach. Future research should prioritize standardized data collection, prospective study designs, and the use of clinically meaningful performance metrics to improve comparability and support robust validation of LOS prediction models.

## 5. Conclusions

This SR summarizes results from five studies that applied AI models to predict postoperative hospital LOS in patients undergoing H&N cancer surgery. Although all studies employed supervised learning approaches and considered clinically relevant variables such as age, comorbidities, and surgical details, the predictive performance and selected features varied substantially. Despite the relatively reliable results of the predictive models, with accuracies ranging from moderate to good (0.63–0.84) and AUROC values between 0.66 and 0.80, indicating variable but overall moderate discriminative performance, significant methodological issues stand out. Importantly, most studies presented a high RoB, primarily due to postoperative predictors incompatible with real-time clinical application, inadequate reporting of model performance metrics, reliance on univariable predictor selection, and failure to account for overfitting and optimism in model performance. Additionally, further limitations were their retrospective and single-center designs, absence of external validation, and inadequate handling of missing data or sample splits. These limitations undermine the generalizability and real-world applicability of the models.

Overall, while AI has the potential to enhance care planning in H&N cancer surgery, methodological flaws limit the current evidence. Future research should prioritize prospective, multicenter studies with standardized data collection, appropriate validation strategies, and transparency in model development to generate robust and clinically useful prediction tools.

Prospective, multicenter studies with standardized variables and clinically relevant evaluation metrics are essential to enable reliable external validation and clinical translation of AI-based LOS prediction models.

## 6. Other Information

### Protocol and Registration

The present SR was conducted following the guidelines of Preferred Reporting Items for Systematic Reviews and Meta-Analysis (PRISMA) [14,28] and the PRISMA-P [26,29] checklist, which is registered at the International Prospective Register of Systematic Reviews (PROSPERO) database under protocol number CRD420251039985.

## 7. Declaration of Generative AI and AI-Assisted Technologies in the Writing Process

During the preparation of this work the authors used ChatGPT (Mar 14 version) from OpenAI (https://chat.openai.com/chat) to specifically review grammar and spelling. After using this tool/service, the authors reviewed and edited the content as needed and took full responsibility for the content of the publication. No large language models/tool/service were used to analyze and draw insights from data as part of the research process.

## Figures and Tables

**Table 1:** Characteristics and performance of included studies.

Author. Year (Ref)	Patients	Surgical Context	Input Variables (Summary)	LOS	Feature Selection	AI Models	Performance (Acc/AUROC)	Main Predictors of LOS
Dang et al. 2021 [10]	401	Vestibular schwannoma resection	Demographics, comorbidities, tumor and operative variables	Median 3 days (IQR 3–4)	Stepwise (AIC)	RF, LR	NI	Coronary artery disease, hypertension
Goshtasbi et al. 2020 [4]	2667	Complex H&N surgery	Demographics, labs, ASA, comorbidities, procedure type	10.4 ± 5.5 days	Univariable screening	GLM, ANN, RF, GBM	Acc 0.73–0.76; AUROC 0.66–0.73	Preoperative transfusion, elective surgery, CHF
Liu et al. 2024 [11]	804	H&N free flap reconstruction	Hemodynamics, labs, transfusions, ICU data	Median 10 days (IQR 8–12)	Collinearity + univariable screening	RF, XGBoost	Acc 0.63–0.71; AUROC 0.71–0.80	Smoking, hypertension, albumin, transfusions
Namavarian et al. 2024 [12]	837	Oral cancer surgery	Pre- and intraoperative clinical variables	14.4 ± 6.6 days	Stepwise, LASSO	MVA, LASSO, RF	Acc 0.82–0.84; AUROC NI	Age, creatinine, surgery duration, comorbidities
Vollmer et al. 2023 [13]	300	H&N cancer surgery	Demographics, TNM stage, operative details	29.9 ± 15.7 days	CHAID	XGBoost, SVM, RF, MLP	Acc 0.65–0.81; AUROC NI	Operation time, ischemia time, ASA, ICU stay

ANN: artificial neural network; ASA: refers to the American Society of Anesthesiologists Physical Status Classification System; AUROC: area under the receiver operator characteristics curve; CHAID: Chi-square automatic interaction detection; CHF: congestive heart failure; GBM: gradient boost machine; GLM: generalized linear model; H&N: head and neck; ICU: intensive care unit; LASSO: Least absolute shrinkage and selection operator; LOS: length of stay; LR: logistic regression; MVA: multivariate analysis; NI: not informed; TNM: refers to the classification system developed by the Union for International Cancer Control and the American Joint Committee on Cancer; XGBoost: extreme gradient boosting.

**Table 2:** Risk of Bias across studies.

	RoB				Applicability				
Author/Year (Ref)	Participants	Predictors	Outcomes	Analysis	Participants	Predictors	Outcomes	RoB	Applicability
Dang et al., 2021 [10]	+	−	+	−	+	+	+	−	+
Goshtasbi et al., 2020 [4]	+	+	+	−	+	+	+	−	+
Liu et al., 2024 [11]	+	−	+	−	+	+	+	−	+
Namavarian et al., 2024 [12]	+	−	+	−	+	+	+	−	+
Vollmer et al., 2023 [13]	+	−	+	−	+	+	+	−	+

PROBAST = Prediction model Risk of Bias ASsessment Tool; RoB = risk of bias. * + indicates low RoB/low concern regarding applicability; − indicates high RoB/high concern regarding applicability.

## Data Availability

No new data were created or analyzed in this study.

## Supplementary Materials

The following supporting information can be downloaded at https://www.mdpi.com/article/10.3390/diagnostics16020263/s1, Supplementary Table S1. PICO strategy. Supplementary Table S2. Search strategy. Supplementary Table S3: Excluded articles and reasons for exclusion.
