# Supplementary material for: Artificial Intelligence Approaches to Predict Postoperative Length of Hospital Stay in Head and Neck Cancer Patients: A Systematic Review
Source: Diagnostics (Basel). 2026 Jan 14;16(2):263. doi: 10.3390/diagnostics16020263 (PMC12839552; doi:10.3390/diagnostics16020263)
Supplement: Supplementary file 1 [file diagnostics-16-00263-s001.zip › diagnostics-3960560-supplementary.pdf]

Supplementary Table S1. PICO strategy

**Acronym PICO**	**Keywords**
Patients	“head and neck”
Intervention/Exposition	“prediction model”<br><br>AND<br><br>“artificial intelligence" OR "machine learning" OR "deep learning"
Comparison	Not applicable
Outcome	“length of stay”

Supplementary Table S2. Search strategy

**Database**	**Query**<br>Search date: November 5th, 2024	**Retrieved studies (n)**
Cochrane	length of stay AND artificial intelligence OR machine learning OR deep learning AND head and neck	0
Embase	('length of stay'/exp OR 'length of stay') AND 'prediction model' AND ('artificial intelligence' OR 'machine learning' OR 'deep learning')	225
PubMed	"length of stay"[All Fields] AND "prediction"[All Fields] AND ("artificial intelligence"[All Fields] OR "machine learning"[All Fields] OR "deep learning"[All Fields])	499
Scopus	(TITLE-ABS-KEY ("length of stay") AND TITLE-ABS-KEY ("prediction model") AND TITLE-ABS-KEY ("artificial intelligence" OR "machine learning" OR "deep learning"))	384
Google Scholar	“length of stay” AND “artificial intelligence" OR "machine learning" OR "deep learning" AND “head and neck”	100
ProQuest	title(“length of stay”) AND title(“artificial intelligence" OR "machine learning" OR "deep learning")	96
**TOTAL**		**1,304**

Supplementary Table S3: Excluded articles and reasons for exclusion.

	**Author**	**Title**	**Year**	**Country**	**Journal/Conference name**	**Exclusion Criteria**
1	Dichter et al.	Post-Operative Outcome Predictions in Vestibular Schwannoma Using Machine Learning Algorithms	2024	USA	Journal of Personalized Medicine	Did not predicted the length of stay
2	Mason et al.	Development and Validation of a Multivariable Preoperative Prediction Model for Postoperative Length of Stay in a Broad Inpatient Surgical Population	2023	USA	Surgery	Included other types of cancer/diseases
3	Zaribafzadeh et al.	Development of Multiservice Machine Learning Models to Predict Postsurgical Length of Stay and Discharge Disposition at the Time of Case Posting	2025	USA	Annals of Surgery Open	Included other types of cancer/diseases
4	Jo et al.	Prediction of Prolonged Length of Hospital Stay After Cancer Surgery Using Machine Learning on Electronic Health Records: Retrospective Cross-sectional Study	2021	South Korea	JMIR Medical Informatics	Included other types of cancer/diseases
